# Supplementary material for: The socio-economic and health effects of COVID-19 among rural and urban-slum dwellers in Ghana: A mixed methods approach
Source: PLoS One. 2022 Jul 15;17(7):e0271551. doi: 10.1371/journal.pone.0271551 (PMC9286267; doi:10.1371/journal.pone.0271551)
Supplement: S1 Checklist — (DOCX) [file pone.0271551.s001.docx]

## GRAMMS checklist

**Good Reporting of A Mixed Methods Study (GRAMMS)**

| **Guideline** | **Section: page** |
| --- | --- |
| Describe the justification for using a mixed methods approach to the research question | Design: Page 7 |
| Describe the design in terms of the purpose, priority and sequence of methods | Design: Pages 7 to 12 |
| Describe each method in terms of sampling, data collection and analysis | Sampling, data collection and analysis: Pages 7-12 |
| Describe where integration has occurred, how it has occurred and who has participated in it | Results: Pages 7, 12-31  Discussion section: Pages 32-37  Conclusion: Pages 39 and 40 |
| Describe any limitation of one method associated with the present of the other method | Limitations: Pages 40 and 41 |
| Describe any insights gained from mixing or integrating methods | Discussion and conclusion: Pages 33-40 |
