# Supplementary material for: The socio-economic and health effects of COVID-19 among rural and urban-slum dwellers in Ghana: A mixed methods approach
Source: PLoS One. 2022 Jul 15;17(7):e0271551. doi: 10.1371/journal.pone.0271551 (PMC9286267; doi:10.1371/journal.pone.0271551)
Supplement: S1 File — (DOCX) [file pone.0271551.s002.docx]

**IDI guide for community leaders (chiefs, queen mothers, herbalists, religious leaders, elders)**

**Title of study: The socio-economic and health effects of COVID-19 among vulnerable populations: Evidence from rural and urban slum dwellers in the ashanti and Volta Regions of Ghana.**

**Background characteristics of Respondent**

**Community name**

Age, sex, education, occupation, position in the community

**Knowledge of COVID-19, preventive measures and how it is shaping community interactions**

1. How did community members learn about COVID-19?
   1. Probe for interaction from the local government, health institutions
   2. Probe for type of engagement such as media
   3. Probe for frequency of information received on COVID-19
   4. Probe for content of information received
   5. How are community members dealing/processing information that they have received from the mentioned sources?
2. What do community members perceive COVID-19 to be?
3. How are community members protecting themselves from COVID-19?
4. How did community members learn to prevent it?
5. What support have you received from the government in your efforts at preventing COVID-19?

**Most Vulnerable groups in communities**

1. Who are the most vulnerable in this community in terms of the ability to prevent themselves from contracting COVID-19?
2. How have persons with disabilities been supported to prevent themselves from being infected with COVID-19?

**Communities as partners**

1. What needs are the most important for your community to be able to prevent yourselves from getting COVID-19?
2. Probe for food, WASH, the ability to seek employment
3. Which external bodies have been supporting your community in the fight against COVID-19
4. What is the form of support that they have been offering you? (Probe for services offered, donation etc)
5. How is the support offered?
6. How are you involved in the in the processes leading to the support being offered?
7. How are you supporting your community in the prevention of the spread of COVID-19?

**Communities owning the process of transformation**

1. How do you think you can continue to protect yourself from COVID-19 and other infections?
2. What are the traditional strategies to protecting yourself from COVID-19?
3. How do you think you can protect yourself from such future occurrences?

**Community health Volunteers**

1. What is the role of community health workers in this community?
2. How do they engage with community members on matters of health?
3. How do you think they are contributing to providing information to community members on COVID-19 prevention?

**Recommendations**

1. What do you think is the best way for the government and other institutions to engage with your community on COVID-19 and other related issues?
2. How do you think the message should be carved to ensure that all community members understand and abide by it?
3. What do you think can be done to improve information and education on COVID-19 in your community?
   1. How can external bodies engage with you well on such matters?
   2. How can community leaders contribute to such a process?
   3. What about community members, how will community members be able support such a process?

**The interview has ended. Thank you very much for participating**
