# Supplementary material for: The socio-economic and health effects of COVID-19 among rural and urban-slum dwellers in Ghana: A mixed methods approach
Source: PLoS One. 2022 Jul 15;17(7):e0271551. doi: 10.1371/journal.pone.0271551 (PMC9286267; doi:10.1371/journal.pone.0271551)
Supplement: S2 File — (DOCX) [file pone.0271551.s003.docx]

**Interview Guide for Community Members**

**Title of study: The socio-economic and health effects of COVID-19 among vulnerable populations: Evidence from the Ashanti and Volta Regions of Ghana**

**Community name:……………………..**

**Background characteristics of Respondent**

Age, sex, education, occupation

**Knowledge on COVID-19 and preventive measures**

1. What do you know about COVID-19?
2. Where did you learn about it?
3. How do community members protect themselves from contracting COVID-19?
4. How do you protect yourself from getting infected with COVID-19?
   1. What about your household members?
   2. Who taught you to prevent it?

**COVID-19 intervention measures’ effect on economic wellbeing of community members**

1. How has COVID-19 affected your work?
   1. Probe for returns in terms of income, customers or workload
2. What have you been doing to ensure that you are able to retain your work?
3. How are you able to take care of your basic needs now that there is COVID-19?
4. Who are the people supporting you to meet your basic needs?
5. What about your dependents, how are you able to take care of them?

**COVID-19’s influence on the socio-cultural aspects of community life**

1. How has COVID-19 affected interactions in your household?
   1. What about your interactions with members outside your household?
   2. What about community relations?
2. Who has been supporting community members in the prevention of COVID-19 infections?
   1. Probe for government support and the nature of the support
   2. How adequate is the support?
   3. Probe for other bodies who are supporting and the kinds of support being offered
3. How are community leaders helping community members to prevent themselves from getting infected with COVID-19? Probe the following:
   1. What forms of support are they offering community members?
   2. How adequate is the support being offered by community leaders?
4. Where did community members seek health care prior to the advent of COVID-19?
5. How has COVID-19 affected community members’ health care seeking behaviour?
   1. Why do you think it has affected community members’ ability to seek health care?
6. What about you, how has COVID-19 influenced your health care seeking behavior?

**Coping strategies and recommendations**

1. How has COVID-19 changed your life?
   1. Probe for positive and negative aspects, social relations, economic, spiritual
2. How can you be supported to prevent COVID-19 in your household?
3. How can the government support the community with the right information to help members protect themselves and their families from COVID-19?
4. How can community leaders support community members to protect themselves from COVID-19?
5. What about you, what can you do to improve your efforts aimed at preventing yourself from COVID-19 infection?

**The interview has ended. Thank you very much for participating**
