# Supplementary material for: The socio-economic and health effects of COVID-19 among rural and urban-slum dwellers in Ghana: A mixed methods approach
Source: PLoS One. 2022 Jul 15;17(7):e0271551. doi: 10.1371/journal.pone.0271551 (PMC9286267; doi:10.1371/journal.pone.0271551)
Supplement: S3 File — (DOCX) [file pone.0271551.s004.docx]

**Observation Checklist**

**Title of study: The socio-economic and health effects of COVID-19 among vulnerable populations: Evidence from rural and slum dwellers in the ASHANTI AND Volta RegionS of Ghana.**

**Observation check list**

**Part 1**

Transect walk to find out important locations in the community

**Part 2**

**Community preparedness**

- Are there WASH equipment dotted around the community?
- Are there posters on COVID-19 pasted in public places?
- Where do community members hang out?
- What are interactions in communities about?

**Interpersonal interactions**

- How are people interacting in the communities?
- Are people using the protocols such as:
  - Nose mask
- Washing of hands
- Using sanitizers
- Observing social distance

**Household level**

- What is the medium of communication on national issues?
- Do households own radios?
- Do they listen to radio talks?
- What times are the radio talks aired?
  - Who listens to them?
- Are there persons with disabilities in households?
- Who communicates COVID-19 information to persons with disability (blind, deaf, crippled)

How are they being protected from contracting COVID-19?
