# Supplementary material for: The socio-economic and health effects of COVID-19 among rural and urban-slum dwellers in Ghana: A mixed methods approach
Source: PLoS One. 2022 Jul 15;17(7):e0271551. doi: 10.1371/journal.pone.0271551 (PMC9286267; doi:10.1371/journal.pone.0271551)
Supplement: S1 Questionnaire — (DOCX) [file pone.0271551.s005.docx]

**Survey Questionnaire**

**Questionnaire on “The socio-economic and health effects of COVID-19 among vulnerable populations: Evidence from the Ashanti and Volta Regions of Ghana’’**

**Demographic Characteristics**

1. Age

a. 18-32 years [1] b. 33-46 years [2] c. 47-60 years [3] d. 61 years and above [4]

2. Place of residence

a. Rural [ 1] b. Slum [2]

3. Sex

a. Male [1] b. Female [2]

4. Religion

a. Christianity [1] b. Islam [2] c. Traditionalist [ 3] d. Other (Please specify [4])………..

e. None [5]

5. Marital status

a. Married [1] b. Single [2] c. Divorced [3] d. Widowed [4] e. Separated [5] f. Cohabitating [6]

6. Educational qualification (highest)

a. Basic [ 1 ] b. Secondary [ 2] c. Tertiary [ 3 ] d. None [4]

7. Employment Status

a. Employed [1] b. Unemployed [2]

8. If employed, please what is your occupation?

a. Trader [1] b. Farmer [2] c. Artisan [3] d. Civil Servant [4] e. Teacher [ 5 ] f. Other (Please specify [6])………..

9. What is your monthly income?

a. GHC10-GHC100 [1] b. GHC101-GHC500 [2] c. GHC501-GHC1000 [3] d. GHC1001 and above [4].

**Awareness and Knowledge on COVID-19**

10. How did you first hear of COVID-19?

a. Relative/friend [1] b. Radio/Television [ 2] c. Health Personnel [3 ] d. Social Media [4 ] e. Other (Please specify [5])………..

11. Do you normally get daily updates on COVID-19?

a. Yes [1] b. No [2]

12. If yes to Q11, please where do you normally get this update from?

a. Relative/friend [1] b. Radio/Television [2] c. Health Personnel [3] d. Social Media [4] e. Other (Please specify [5])………..

13. Please what is the incubation period of the coronavirus?

a.1-6 days [ 1 ] b. 7-12 days [ 2 ] c. 14 days and above [ 3 ] d. don’t know [ 4 ]

14. COVID-19 can be transmitted from one person to another through saliva and nasal droplets as a result of talking, singing, coughing and sneezing?

a. Yes [1] b. No [2] c. Don’t know [3]

15. COVID-19 can be transmitted from one person to another by shaking hands and hugging?

a. Yes [1] b. No [2] c. Don’t know [3]

16. COVID-19 can be transmitted through infected aerosols?

a. Yes [1] b. No [2] c. Don’t know [3]

17. COVID-19 can be prevented through frequent hand washing with soap under running water and the use of hand sanitiser?

a. Yes [1] b. No [2] c. Don’t know [3]

18. COVID-19 can be prevented by maintaining 1 meter physical distance from others?

a. Yes [1] b. No [2] c. Don’t know [3]

19. COVID-19 can be prevented by wearing face and nose masks, especially in crowded places?

a. Yes [1] b. No [2] c. Don’t know [3]

**COVID-19 Attitudes, Practices and Associated Barriers**

Please kindly rate how often you adhere to the following COVID-19 protocols.

**[1 –Not at all; 2 - Sometimes; 3 - Often; 4 - Always]**

|  |  | **1 2 3 4** |
| --- | --- | --- |
| 20 | Wearing of face masks in crowded or public places | ☐ ☐ ☐ ☐ |
| 21 | Maintaining 1 meter physical distance from others | ☐ ☐ ☐ ☐ |
| 22 | Frequent hand washing with soap under running water | ☐ ☐ ☐ ☐ |
| 23 | Using hand sanitisers frequently in the absence of running water and soap | ☐ ☐ ☐ ☐ |
| 24 | Going out only when it is needed | ☐ ☐ ☐ ☐ |

Please kindly rate how the following hamper your adherence to COVID-19 protocols.

**[1 –Strongly disagree 2-Disagree 4 –Agree 3 – Strongly Agree]**

|  |  | **1 2 3 4** |
| --- | --- | --- |
| 25 | Wearing face mask makes me feel uncomfortable | ☐ ☐ ☐ ☐ |
| 26 | I do not have a face mask | ☐ ☐ ☐ ☐ |
| 27 | No access to running water in dwelling | ☐ ☐ ☐ ☐ |
| 28 | No access to toilet facility in dwelling |  |
| 29 | No money to buy soap | ☐ ☐ ☐ ☐ |
| 30 | Cannot find hand sanitisers to buy |  |
| 31 | No money to buy hand sanitisers | ☐ ☐ ☐ ☐ |
| 32 | I feel uncomfortable using hand sanitisers frequently | ☐ ☐ ☐ ☐ |
| 33 | I cannot stay home without meeting my friends/relatives to chat | ☐ ☐ ☐ ☐ |
| 34 | I think that COVID-19 is for rich people | ☐ ☐ ☐ ☐ |
| 35 | I think that COVID-19 is not real | ☐ ☐ ☐ ☐ |

**Socio-Economic and Health Effects of COVID-19**

Please kindly rate how COVID-19 has affected the following aspects of your life.

**[1 –Strongly disagree 2-Disagree 4 –Agree 3 – Strongly Agree]**

|  |  | **1 2 3 4** |
| --- | --- | --- |
| 36 | It has negatively affected my job | ☐ ☐ ☐ ☐ |
| 37 | My income has fallen | ☐ ☐ ☐ ☐ |
| 38 | The prices of food have increased | ☐ ☐ ☐ ☐ |
|  | I am unable to afford enough food due to rising prices |  |
| 39 | I am afraid to seek healthcare from a health facility even when I am unwell | ☐ ☐ ☐ ☐ |
| 40 | It makes feel depressed | ☐ ☐ ☐ ☐ |
| 41 | It makes me anxious | ☐ ☐ ☐ ☐ |
