# Supplementary material for: The socio-economic and health effects of COVID-19 among rural and urban-slum dwellers in Ghana: A mixed methods approach
Source: PLoS One. 2022 Jul 15;17(7):e0271551. doi: 10.1371/journal.pone.0271551 (PMC9286267; doi:10.1371/journal.pone.0271551)
Supplement: Attachment — Submitted filename: Response to Editor and reviewers.docx [file pone.0271551.s006.docx]

**Response to Editor’s comments**

1. Please ensure that your manuscript meets PLOS ONE's style requirements, including those for file naming.

**Response: Authors have ensured that the manuscript has met the PLOS ONE requirements, including those for file naming**

2. Please state what role the funders took in the study. If the funders had no role, please state

**Response: The funders had no role in study design, data collection and analysis, decision to publish, or preparation of the manuscript.**

3. We note that you have indicated that data from this study are available upon request. PLOS only allows data to be available upon request if there are legal or ethical restrictions on sharing data publicly. For more information on unacceptable data access restrictions, please see http://journals.plos.org/plosone/s/data-availability#loc-unacceptable-data-access-restrictions. In your revised cover letter, please address the following prompts:
   a. If there are ethical or legal restrictions on sharing a de-identified data set, please explain them in detail (e.g., data contain potentially sensitive information, data are owned by a third-party organization, etc.) and who has imposed them (e.g., an ethics committee). Please also provide contact information for a data access committee, ethics committee, or other institutional body to which data requests may be sent.

**Response: There are ethical restrictions on sharing the data.**

**The address is the Administrator, Research Ethics Committee University of Health and Allied Sciences, PMB 31, Ho, Volta region. Email: rec@uhas.edu.gh**

b. If there are no restrictions, please upload the minimal anonymized data set necessary to replicate your study findings as either Supporting Information files or to a stable, public repository and provide us with the relevant URLs, DOIs, or accession numbers. For a list of acceptable repositories, please see <http://journals.plos.org/plosone/s/data-availability#loc-recommended-repositories>. We will update your Data Availability statement on your behalf to reflect the information you provide.

**Response: There are ethical restrictions**

**Response: Thank you**

4. Your ethics statement should only appear in the Methods section of your manuscript. If your ethics statement is written in any section besides the Methods, please move it to the Methods section and delete it from any other section

**Response: Ethics statement included in methods section, please see lines 254-263**

5. Please include captions for your Supporting Information files at the end of your manuscript, and update any in-text citations to match accordingly.

**Response: Captions for supporting information files included at the end of the manuscript, please see lines 792 -797**

**-** **Reviewer #1:**

**Thank you very much, for your review. Please, find below the responses to your comments**

Major comments

-English language requires further improvement. Some sentences are difficult to understand and may require rephrasing.

**Response: English language improved in all sections of the manuscript.**

-Include latitude/longitude coordinates of the study area in methods section.

**Response: latitude/longitude coordinates of the study area in methods section, kindly see lines 132 and 133 and 139**

-The materials and methods section should be sub-divided into sections such as Study setting, Study design and population, Sample size and sampling procedure, Data collection, Quality control and assurance, Data management and analysis, and Ethical considerations.

**Response: Study setting, Study design and population,** **Sample size and sampling procedure, Data collection, Quality control and assurance, Data management and analysis, included for quantitative and qualitative methods. Kindly see lines 123 -253**

**Response” Ethical considerations and ethics approval number included, kindly see lines 255-263**

**Minor comments**

-The expansion of COVID-19 is "Coronavirus disease 2019 (COVID-19)"

**Response: Coronavirus disease 2019 (COVID-19) corrected. Kindly see line 77**

-Mention all $172915600 value in millions rather than full numbers.

**Response: Value changed to millions rather than full numbers. Kindly see lines 92 and 94**

**Reviewer #2: Thank you for your insight, very useful comments and commendation.**

**Response to comments**
Introduction: Please kindly provide the complete picture and data regarding the prevalence of COVID-19 at rural and urban-slum dwellers in Ghana as well as the impact of COVID-19 to these settings.

**Response:** **The prevalence of COVID-19 in the study regions have been reported, kindly see lines see 89-91. COVID-19 cases are reported by administrative regions, Ghana does not record prevalence rates by rural and urban settlements, so we are unable to provide such data.**

**Response: The impact of COVID-19 on rural and urban-slum dwellers in Ghana reported. Kindly see lines 108-116.**

Study design: Cite and elaborate any mixed methods approach that you used on this study.

**Response: Mixed methods approach cited and elaborated, kindly see lines 145-153**

Study area: You should mention the prevalence or any data of the study areas in the introduction part. Was Adaklu city also having high prevalence compared to other rural areas?

**Response: The prevalence or any data of the study regions have been reported, kindly see lines 90 & 91. The reason for the inclusion of Adaklu district in the study has been explained, kindly see lines 134-142.**

Sampling technique: Elaborate more on how to randomize your sample

**Response: Elaboration of randomized sample explained, kindly see lines 166-169.**

Please explain on how to establish rigour and trustworthiness of mixed methods data.

**Response: Rigour and trustworthiness of the study have been reported in the methodology section. Also, the** **GRAMMS checklist was used and has been included as Supporting Information. Kindly see lines 151 and 152**

Conclusion: Please make a concise, clear and short conclusion and policy implications.

**Response: a concise, clear and short conclusion and policy implications has been included, kindly see lines 727-755**

Ethics statement: Give the number and year of ethics approval.

**Response: Please, the number and year of ethics approval have been reported in lines 255-256.**
